# 

**Published:** 1880-03

**Authors:** 


*CONTENTS.*

| | Page |
|---|---|
| The Nature of Cough | 65 |
| Physical Education of Children | 66 |
| How to Keep Cool | 68 |
| The First Sign of Consumption | 68 |
| Summer Complaint | 69 |
| Clean Your Cellars | 70 |
| Shaving the Beard | 72 |
| Ventilation | 73 |
| To Stop Coughing | 75 |
| Bathing | 77 |
| Hot Air Furnaces | 78 |
| Warts | 79 |
| Hunger | 79 |
| Ignorance and Health | 80 |
| Rage and Ruin | 80 |
| Valuable Table | 81 |
| Incurable Insanity | 81 |
| Consumption—A Suggestion | 82 |
| Longevity | 83 |
| Cleanliness | 84 |
| A Good Wife | 84 |
| Cause of Death | 85 |
| Be Kind to Children | 86 |
| Popular Fallacies | 87 |
| Flower Pots | 88 |
| Spirits | 89 |
| Dying Words of Celebrated Persons | 92 |
| Architects and Bath-Rooms | 93 |

